# L1198F Mutation Resensitizes Crizotinib to ALK by Altering the Conformation of Inhibitor and ATP Binding Sites

**DOI:** 10.3390/ijms18030482

**Published:** 2017-02-24

**Authors:** Jian Li, Rong Sun, Yuehong Wu, Mingzhu Song, Jia Li, Qianye Yang, Xiaoyi Chen, Jinku Bao, Qi Zhao

**Affiliations:** 1 School of Medicine, Chengdu University, Chengdu 610106, China; lijian01@cdu.edu.cn (J.L.); songmingzhu@cdu.edu.cn (M.S.); lijia0619@foxmail.com (J.L.); 2 College of Life Sciences and Key Laboratory for Bio-Resources of Ministry of Education, Sichuan University, Chengdu 610064, China; sunrong198982@163.com; 3 School of Information Science and Engineering, Chengdu University, Chengdu 610106, China; yuehongwu.cdu@foxmail.com; 4 Sichuan Industrial Institute of Antibiotics, Chengdu University, Chengdu 610052, China; qianyeyang@foxmail.com; 5 College of Pharmacy and Biological Engineering, Chengdu University, Chengdu 610106, China; xiaoyichen.cdu@foxmail.com

**Keywords:** ALK, point mutation, drug resistance, resensitization, lorlatinib

## Abstract

The efficacy of anaplastic lymphoma kinase (ALK) positive non-small-cell lung cancer (NSCLC) treatment with small molecule inhibitors is greatly challenged by acquired resistance. A recent study reported the newest generation inhibitor resistant mutation L1198F led to the resensitization to crizotinib, which is the first Food and Drug Administration (FDA) approved drug for the treatment of ALK-positive NSCLC. It is of great importance to understand how this extremely rare event occurred for the purpose of overcoming the acquired resistance of such inhibitors. In this study, we exploited molecular dynamics (MD) simulation to dissect the molecular mechanisms. Our MD results revealed that L1198F mutation of ALK resulted in the conformational change at the inhibitor site and altered the binding affinity of ALK to crizotinib and lorlatinib. L1198F mutation also affected the autoactivation of ALK as supported by the identification of His1124 and Tyr1278 as critical amino acids involved in ATP binding and phosphorylation. Our findings are valuable for designing more specific and potent inhibitors for the treatment of ALK-positive NSCLC and other types of cancer.

## 1. Introduction

Lung cancer is one of the most life-threatening malignancies worldwide and is the leading cause of cancer-related death for both men and women [1]. Anaplastic lymphoma kinase (ALK) is a member of the receptor tyrosine kinases (RTKs), which belong to the insulin receptor kinase superfamily [2]. Chromosomal rearrangements in the *ALK* gene lead to the deregulation of ALK kinase activity, which in turn alters the downstream signaling pathways in cancer biology [3]. Abnormal expression of fused ALK genes has been implicated in the pathogenesis of several types of cancer, including non-small-cell lung cancer (NSCLC), anaplastic large-cell lymphoma, glioblastoma, and neuroblastoma [4]. Despite the fact that ALK rearrangement only occurs in 3%–7% of NSCLC patients, its total number of cases is larger than those of several other malignancies [5].

Inhibition of deregulated kinase activities by small molecule inhibitors has been proven to be an effective treatment for many types of diseases, including chronic myeloid leukemia [6], epidermal growth factor receptor (EGFR)-mutated [7,8], and ALK-rearranged NSCLC [9]. Crizotinib is the first ALK inhibitor to treat NSCLC approved by the Food and Drug Administration (FDA)-approved ALK inhibitor to treat NSCLC, which has a classical ATP-competitive mechanism of action [3]. Although crizotinib has demonstrated itself as an efficient counter to ALK rearranged NSCLC, acquired resistance developed quickly after its launch has made its beneficial effects temporary. Mutation-driving drug resistance has emerged as a major roadblock for the development of targeted small molecule inhibition for cancer treatment [10]. The principal mechanisms of acquired crizotinib resistance include secondary resistance mutations in the kinase domain of ALK, for example, L1196M, the ‘gate-keeper’ mutation and the C1156Y mutation [11]. Currently, the practical way to overcome such resistance is to treat the patients with more potent and selective next-generation inhibitors [12,13,14,15,16]. A number of newer generation ALK inhibitors have been developed, including ceritinib, alectinib, brigatinib, and lorlatinib, to overcome resistance caused by mutations in the ALK protein [15,16,17].

Molecular dynamics (MD) simulation is a computational technique that has been widely used to obtain information on the time evolution of conformations of proteins and other biological macromolecules and also kinetic and thermodynamic information [18,19]. Studying the interaction and binding patterns of the drug with MD at the molecular level helps us understand the mechanism of the drug action and has proven to be a significant part of drug design [20,21]. Molecular dynamics measures the change of confirmation at picosecond time intervals, which enables us to understand instability and loss of interaction caused by mutations, as well as their adverse effects on the drug metabolism [20]. Recently, Shaw et al. described an intriguing case of ALK inhibitors resistance [22]. L1198F mutation on the fused ALK protein resensitized a patient who had the gatekeeper C1156Y mutation to crizotinib—the first generation ALK inhibitor. Clinically, it is extremely rare to see a cancer mutate to become resensitized to an older generation of targeted therapy. Understanding the molecular mechanism behind these changes of drug sensitivity is of great importance to the design of the newer generations of ALK inhibitors. In this study, we took the MD approach to dissect the molecular mechanism behind this event. Our results provide valuable information for the design of more specific and effective treatment of ALK rearranged NSCLC and other types of cancer.

## 2. Results and Discussion

### 2.1. Root-Mean-Square Deviation Analysis of the Protein Backbones in Crizotinib/Lorlatinib Associated ALKs

**Figure 1:**
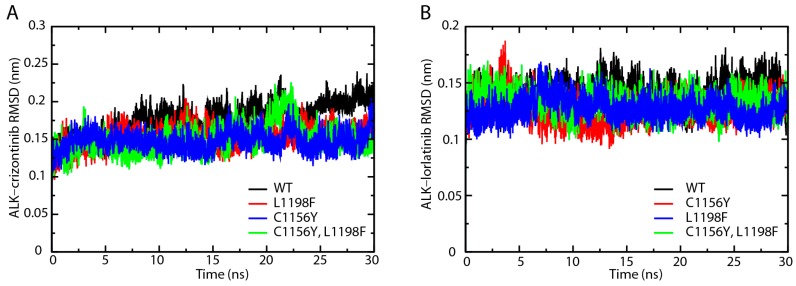
Root-mean-square deviation (RMSD) analysis of crizotinib/lorlatinib associated mutated anaplastic lymphoma kinase (ALK). (**A**) Wild type (WT), C1156Y, L1198F, and C1156Y-L1198F ALK protein associated with crizotinib; (**B**) Wild type (WT), C1156Y, L1198F, and C1156Y-L1198F ALK protein associated with lorlatinib.

We performed molecular dynamics simulation of the ALK-inhibitor complexes for 30 ns with GROMACS software. We first analyzed the root-mean-square deviation (RMSD) of the protein backbones in crizotinib or lorlatinib associated wild type, C1156Y, L1198F, and C1156Y-L1198F mutants. As shown in Figure 1A, the RMSD of ALK–crizotinib complexes quickly reached a steady state after 5 ns of simulation. The fluctuation of the wild type ALK was slightly higher than the other mutants. The C1156Y-L1198F mutant experienced a leap of RMSD up to 0.2 nm from around 20 ns to 25 ns. Up to the end of the 30 ns simulation, the RMSD of C1156Y, L1198F, and C1156Y-L1198F mutants were steady around 0.15 nm, while the value of the wild type protein was moderately higher than the others mutants. The RMSD of ALK mutants complexed with lorlatinib were fairly stable throughout the whole course of simulation (Figure 1B). There was no significant difference among the protein backbones analyzed. According these results, crizotinib or lorlatinib association did not significantly affect the RMSD protein backbones.

### 2.2. Crizontinib and Lorlatinib Binds to ALK (C1156Y-L1198F) with Different Affinity

**Figure 2:**
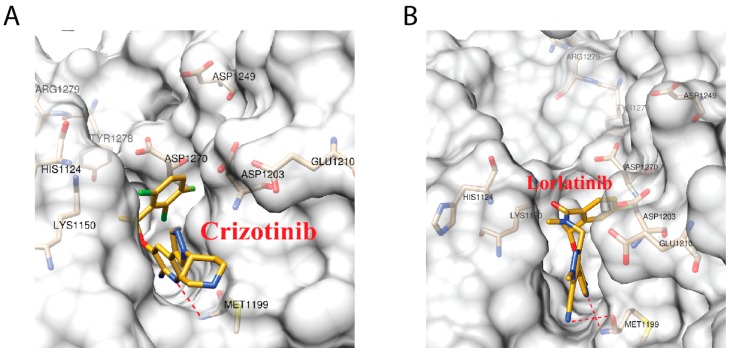
ALK–crizotinib (panel **A**, PDB ID: 5AAB) and ALK–lorlatinib (panel **B**, PDB ID: 5AA8) complexes structures. The ligands and key amino acid residues of ALK are presented as stick models. The dashed red lines represent the hydrogen bonds predicted by Ligplot+.

In order to elucidate the mechanism that rendered the resensitization of ALK (C1156Y-L1198F) to crizotinib, we decided to focus on comparing ALK–crizotinib and ALK–lorlatinib complexes. We will refer to ALK (C1156Y-L1198F) double mutants simply as ALK in the future discussion. Structurally, crizotinib and lorlatinib bond to the same pocket of ALK protein (Figure 2A,B). The major difference is that lorlatinib is supposed to have a higher selectivity, which is achieved by the targeting of L1198 presented in only 25% of the kinases.

**Figure 3:**
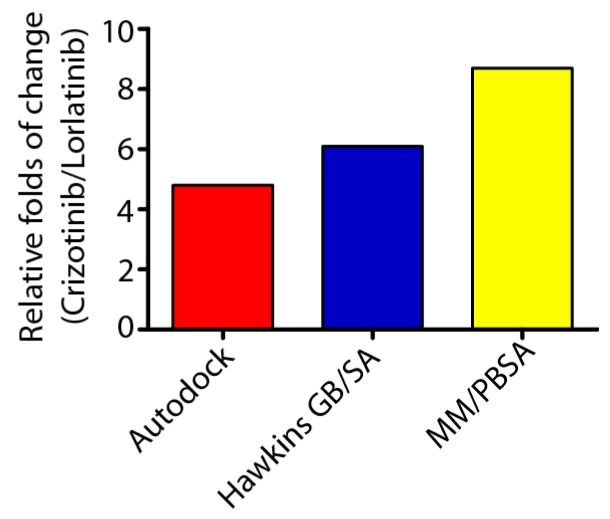
Energy decomposition of ALK–crizotinib and ALK–lorlatinib complexes. The binding energy of crizotinib and lorlatinib to ALK were calculated by AutoDock Vina 1.1.2, Hawkins generalized Born surface area (Hawkins GB/SA), and molecular mechanics Poisson–Boltzmann surface area (MM/PBSA) methods. The relative folds of energy change were defined as the folds of binding energy decrease from lorlatinib to crizotinib.

Binding energy analysis of ALK–inhibitor complex by AUTODOCK software revealed that crizotinib binds to ALK with a much lower energy (25.9 kJ/mol) compared with lorlatinib (123.7 kJ/mol) (Table S1). This data indicated that crizotinib bound to ALK protein tighter than lolatinib. The prediction was in line with the inhibition constant (Ki) and half-maximal inhibitory concentration (IC_50_) values presented by Shaw et al. [22]. Further, energy decomposition by Hawkins generalized Born surface area (Hawkins GB/SA), molecular mechanics Poisson–Boltzmann surface area (MM/PBSA) binding energy analysis also supported this conclusion (Table S1). As shown in Figure 3, the predicted electrostatic energy of ALK–crizotinib complex using the above-mentioned methods were 4.8-, 6.1-, and 8.7-folds lower than the ALK–lorlatinib complex, suggesting a higher affinity of crizotinib to the ALK (C1156Y-L1198F) mutant protein than lorlatinib.

### 2.3. Root-Mean-Square Deviation Comparison of ALK–Crizotinib and ALK–Lorlatinib Complexes

**Figure 4:**
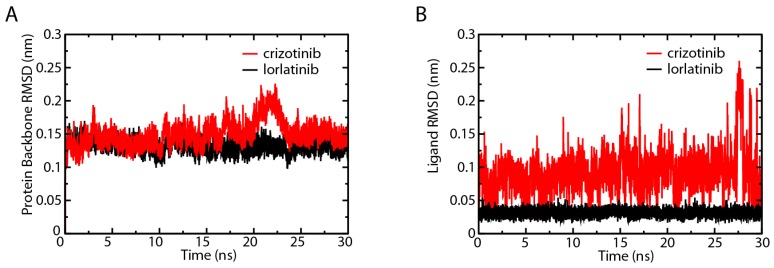
Root-mean-square deviation (RMSD) comparison of the ALK–crizotinib and ALK–lorlatinib complexes Root-mean-square deviationsof the protein backbones (**A**); and ligands (**B**) in ALK–crizotinib and ALK–lorlatinib complexes were calculated and compared.

ALK protein in the ALK–crizotinib and ALK–lorlatinib complexes were comparably stable during the whole course of 30 ns simulation, except for that ALK–crizotinib complex fluctuated during 20–25 ns (Figure 4A). Since this fluctuation only lasted for a relatively short period of time, it might result from the adjustment of the binding pocket of ALK to the crizotinib in the process of protein–ligand association. This was supported by the RMSD analysis of the two ligands. We found that the RMSD of crizotinib was fluctuating violently throughout the course of simulation (Figure 4B), indicating that the drug binding pocket of ALK was reorganized considerably upon the association of crizotinib. Such reorganization was confined to the binding pocket of crizotinib, because the ALK protein as a whole remained relatively steady. The RMSD of lorlatinib was extremely stable from the start to the end (Figure 4B). Generally, a narrower fluctuation of the RMSD means the system is more stable. The RMSD analysis results strongly indicated that the binding affinity change resulted from L1198F mutation was not enough to explain the observed drug sensitivity shift. The deregulation of the other key events, such as ATP association and the substrate phosphorylation, might also contribute to the change of drug sensitivity.

### 2.4. Identification of the Key Amino Acid Residues Affecting ALK Activity

**Figure 5:**
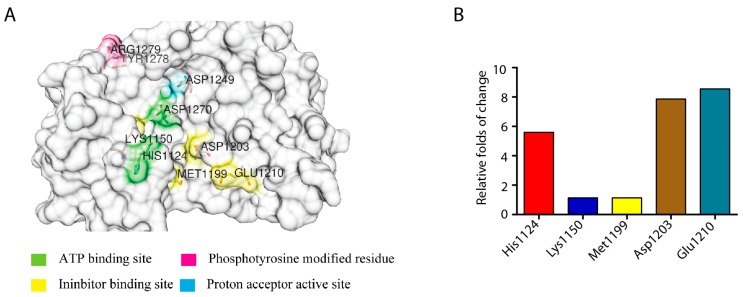
Amino acid residues affecting ALK activity. (**A**) Key amino acid residues affecting ALK activity identified by analyzing the trend of electrostatic energy change (PDB ID: 5AA8); (**B**) Relative folds of energy change of the identified key amino acid residues.

In order to determine the key amino acid residues that are affecting the ALK activity upon inhibitor binding, we investigated the electrostatic energy change trends of ALK–crizotinib and ALK–lorlatinib complexes for three periods of time in MD simulation (5–10 ns, 20–25 ns, 25–30 ns) (Table S2). Only the amino acid residues that kept the same trend of energy change, either constantly increasing or decreasing, were likely to affect the activity of ALK (i.e., contributing to the drug sensitivity change). According to this criterion, we identified 174 amino acid residues (Table S2), among which His1124, Lys1150, Met1199, Asp1203, and Glu1210 were the residues involved in the domains of inhibitor and ATP binding (Figure 5A). We calculated the folds of energy change for the five key residues identified (Table S3). The average folds of the relatively energy change for His1124, Lys1150, Met1199, Asp1203, Glu1210 were 5.60, 1.14, 1.14, 7.86, and 8.55, respectively (Figure 5B).

### 2.5. Crizotinib and Lorlatinib Interacts with ALK in Different Modes

**Figure 6:**
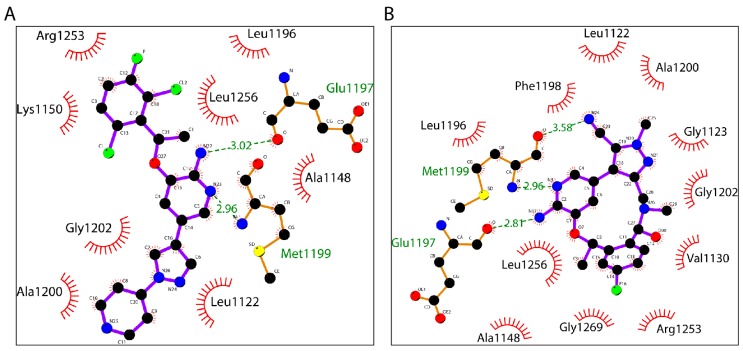
Two-dimensional (2D) interaction diagrams of ALK–inhibitor complexes. The 2D interaction diagrams of crizotinib (**A**, PDB ID: 5AAB) and lorlatinib (**B**, PDB: ID 5AA8) were generated by Ligpolt+ software. Ball and stick denotes ligands. Corresponding ALK residues are shown as wires.

We generated the two-dimensional (2D) interaction diagram of ALK–crizotinib and ALK–lorlatinib to analyze the hydrogen bonds formation and hydrophobic interaction between ALK and the small molecule drug (Figure 6). The N22 and N23 of crizotinib formed two hydrogen bonds with Glu1197 and Met1199 of ALK. The distances of the two hydrogen bonds were 2.96 Å and 3.02 Å, respectively. Lorlatinib interacted with Glu1197 and Met1199 of ALK by forming three hydrogen bonds through the N3, N17, and N24. The distances were 2.96 Å, 2.81 Å, and 3.58 Å, respectively. In terms of hydrophobic interaction, amino acid residues Leu1122, Ala1148, Leu1196, Ala1200, Gly1202, and Leu1256 were conserved between crizotinib and lorlatinib. Lys1150 and Arg1253 were only involved in ALK–crizotinib interaction, while Gly1123, Val1130, Phe1198, and Gly1269 were unique to ALK–lorlatinib interaction.

We also calculated the distance of the inhibitor to the hydrogen bonds forming amino acid residues, Glu1197 and Met 1199, with respect to time (Table 1). During the whole process of simulation, both Glu1197 and Met1199 fluctuated significantly in the ALK–crizotinib and ALK–lorlatinib complexes. This result supported that L1198F mutation caused dramatic conformational changes to the inhibitor binding pocket, especially the amino acid residues nearby.

### 2.6. Root-Mean-Square Fluctuation Analysis of the Key Amino Acid Residues

**Figure 7:**
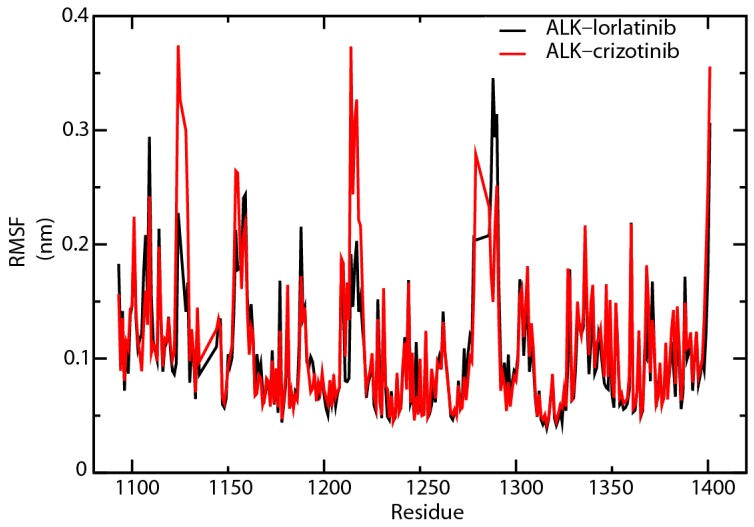
Root-mean-square fluctuation (RMSF) comparison of ALK–inhibitor complexes. Amino acid residues of crizotinib-associated (red line) or lorlatinib-associated (black line) ALK proteins were analyzed for RMSF.

We then analyzed the trends of root-mean-square fluctuation (RMSF) change for the amino acids that participated in ATP binding, inhibitor binding, proton binding, and phosphorylation (Figure 7). The RMSF values of His1124 and Glu1210 in ALK–crizotinib were constantly higher than that of ALK–lorlatinib complex throughout the simulation (Table S4). The average folds of change were 1.80 for His1124 and 1.29 for Glu1210 (Table S4). His1124 is involved in ATP binding and Glu1210 is involved in inhibitor binding, which are the two key events affecting the physiological outcome of treatment targeting ALK.

### 2.7. L1198F Mutation Affects the ATP Association of ALK

**Figure 8:**
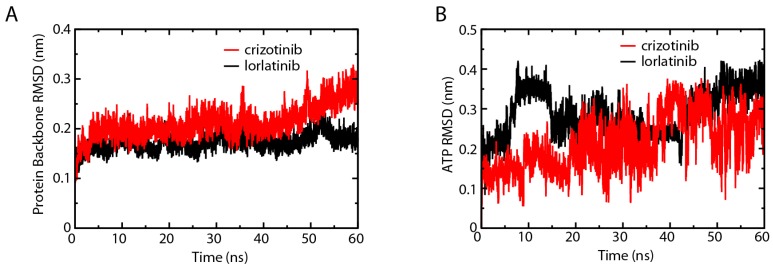
Root-mean-square deviation (RMSD) comparison of ALK–ATP complexes. Root-mean-square deviations of the protein backbones (**A**); and ATP (**B**) in ALK–crizotinib and ALK–lorlatinib complexes were calculated and compared.

Both the electrostatic energy (Figure 5) and RMSF (Figure 7) change trends analysis identified His1124 as a key amino acid involved in regulating the activity of ALK. Since ALK is an abnormally expressed kinase in many types of cancers, it is not surprising to see His1124 that is a key amino acid residue participating in ATP binding functions in the resensitization of crizotinib. A structural study by Bossi et al. suggests that the O2 oxygen of the α-phosphate is within hydrogen-bonding distance of the backbone carbonyl of His1124 of the P-loop [23]. A modeling study of the ALK Gly1123–His1124 segment indicated that mutations in this part of the protein are likely to sterically impede ATP binding [24]. We further analyzed the interaction of ALK and ATP through RMSD and RMSF. Because the inhibitor binding site and the ATP binding site are very close, we were not able to perform a MD simulation of ALK–inhibitor–ATP complex. Instead, we isolated the ALK protein from ALK–crizotinib and ALK–lorlatinib complex and combined them with ATP molecule to run the simulation. The fluctuation of ALK in the crizotinib-associated complex was slightly wider than that of lorlatinib (Figure 8A). The ATP in the two complexes behaved differently (Figure 8B). In the crizotinib-associated complex, the average value of ATP fluctuation was 0.21 nm, in comparison with 0.29 nm of the lorlatinib-associated complex. The largest fluctuation in the crizotinib-associated complex was 0.38 nm, in comparison with 0.42 nm in the lorlatinib-associated complexes. These differences indicated that ATP binding was less stable in the ALK–lorlatinib complex.

**Figure 9:**
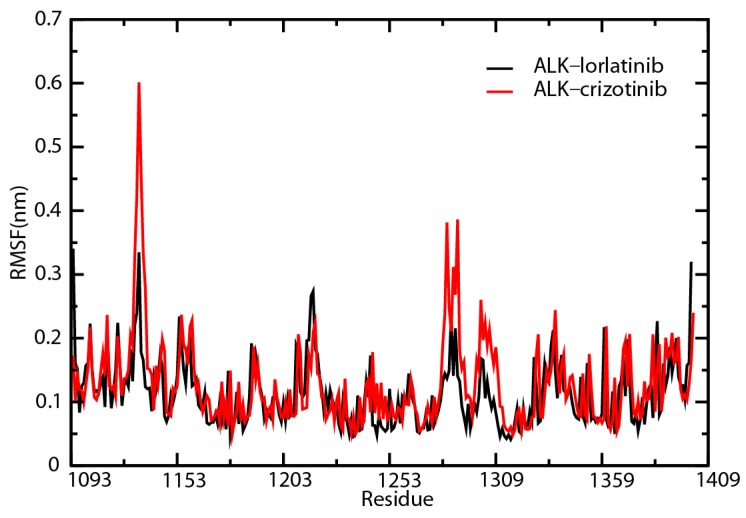
Root-mean-square fluctuation (RMSF) comparison of ALK–ATP complexes. Amino acid residues of crizotinib-associated (red line) or lorlatinib-associated (black line) ALK protein were analyzed for RMSF.

We then analyzed the RMSF change between the two complexes (Figure 9). After 60 ns of simulation, His1124 was identified as the residue that gave the largest RMSF value difference (0.27 nm) between the crizotinib- and lorlatinib-associated ALK protein (Table S5). This result confirmed the importance of His1124 in resensitizing crizotinib to C1156Y-L1198F mutated ALK protein. In addition, the RMSF analysis also found that Arg1279 gave the second largest RMSF value difference (0.24 nm) and Tyr1278 showed a 0.11 nm RMSF difference between the two complexes compared (Table S5). Both Tyr1278 and Arg1279 are in the YRASYY sequence that is present in the activation loop of the kinase domain, and Tyr1278 has been defined as the first tyrosine to be phosphorylated in this sequence [25]. Presumably, the L1198F mutation led to conformational changes in the ATP binding pockets, including His1124. Such changes decrease the binding affinity of ATP and hinder the autoactivation of ALK regulated by the phosphorylation on Tyr1278. These events are likely to affect the downstream phosphorylation of ALK substrates. The critical amino acids identified in our MD analysis, including His1124, Lys1150, Met1199, Asp1203, Glu1210, and Tyr1278, were in line with the experimental results by another group (Table 2). These amino acid residues are involved in inhibitor binding (Lys1150, Met1199, Asp1203, and Glu1210) [24,26], ATP binding (His1124) [24,25], and the phosphorylation (Tyr1278) of the ALK protein [27].

### 2.8. Secondary Structure Analysis of the ATP and Inhibitor Binding Sites

**Figure 10:**
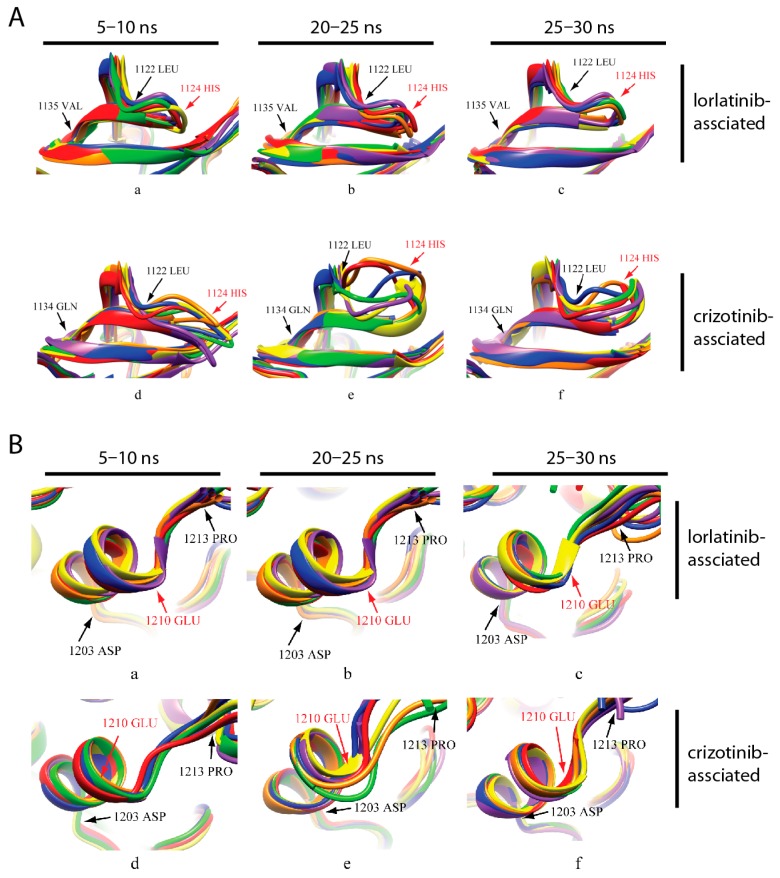
Secondary structure comparison of domains around His1124 and Glu1210. Protein Data Bank (PDB) structures of the inhibitor associated to ALK—lorlatinib-associated (PDB ID: 5AA8; **a**–**c**) and crizotinib-associated (PDB ID: 5AAB; **d**–**f**)—from different time points were extracted and superimposed to analyze the secondary structure change of the domains of around His1124 (**A**) and Glu1210 (**B**). Structures from six different time points combined and color coded (5–10 ns: 5 ns red, 6 ns orange, 7 ns yellow, 8 ns green, 9 ns blue, 10 ns purple; 20–25 ns: 20 ns red, 21 ns orange, 22 ns yellow, 23 ns green, 24 ns blue, 25 ns purple; 25–30 ns: 25 ns red, 26 ns orange, 27 ns yellow, 28 ns green, 29 ns blue, 30 ns purple).

The activity of a protein is often affected by its conformational changes. We analyzed the secondary structure change of the amino acids around His1124 and Glu1210. The secondary structure of the analyzed region (aa1122–1134) was relatively steady in the first 5 ns of the simulation and His1124 was located in a turn (Figure 10A, panels a and d). At 20–25 ns, the structure of the corresponding domain in crizotinib-associated ALK was significantly different from that of the lorlatinib-associated protein (Figure 10A, panels b and e). The change was represented by conversion from turns to coils. This difference was still observable at the end of the simulation (Figure 10A, panels c and f). As shown in Figure 10B, the secondary structure of aa1203–1213 was mainly composed of α-helixes and coils. There was no noticeable difference at residues 1203 to 1213 between ALK–crizotinib and ALK–lorlatinib complexes at the beginning of simulation (5–10 ns). During 20–25 ns, the secondary structure of ALK–crizotinib dramatically shifted from α-helixes to coils (Figure 10B, panels b and f), which was not the case for ALK–lorlatinib. The secondary structure of ALK–lorlatinib remained to be fairly steady throughout the simulation. The predicted secondary structure difference was consistent with the RMSD of crizotinib and lorlatinib, as shown in Figure 4B, indicating dramatic conformational shifts of the ALK protein at the crucial domains to accommodate crizotinib binding.

## 3. Materials and Method

### 3.1. Retrieving the Protein Data Bank Files

The three-dimensional (3D) structures wild type and mutated ALK complexed with inhibitor were downloaded from the Research Collaboratory for Structural Bioinformatics (RCSB) PDB using accession numbers: 2XP2, 4CLI, 5AAA, 5AAB, 5AAC, 5AA8, 5AA9, and 5A9U [26]. The 2XP2, 5AAA, 5AAB, and 5AAC are complexed with crizotinib and 4CLI, 5AA8, 5AA9, and 5A9U are complexed with lorlatinib.

### 3.2. Molecular Docking

The molecular graphics of ALK–inhibitor complexes were prepared and analyzed with the University of California, San Francisco (UCSF) Chimera package [28]. In this process, (i) solvent and non-complexed ions were removed; and (ii) hydrogens and charges (of amber ff99sb force field) were added to the protein [28]. The docking was performed with DOCK 6.5 program (UCSF) under the following parameters: 5AAB, box margin = 0.5 nm, select spheres = 1.0 nm, max orientations = 2300; 5AA8, box margin = 0.5 nm, select spheres = 1.0 nm, max orientations = 5000.

AutoDock Vina analyses were performed in cubes with 1.5 nm side length (5AAB, center_x = 37.935, center_y = 47.567, center_z = 17.016; 5AA8, center_x = 38.053, center_y = 47.101, center_z = 16.983). The side chains of the ligands were allowed for flexible torsion. The maximum energy difference between the optimal energy and the highest energy was set to be 3, and the lowest predicted energies were extracted for further analysis.

The ALK–inhibitor complexes were first analyzed by grid scoring of DOCK 6.5, followed by a second round scoring with Descriptor and Hawkins GB/SA algorithms. Descriptor scoring calculates the standard energy within a system. Hawkins GB/SA scoring calculates the energy through molecular mechanics generalized born surface area (MM/GBSA).

### 3.3. Molecular Dynamics Simulation

Molecular dynamics simulations were performed using GROMACS 4.6.7 [29] package and Amber ff99sb force field with TIP3P water model [30]. Crizotinib and lorlatinib were under the general Amber force field (GAFF) and the charges were added by AM1-BBC method. We performed system check with parmchk program and generated additional parameters of force field. To generate the ligand topology file, we used AnteChamber PYthon Parser interfacE (ACPYPE) [31]. Particle Mesh Ewald (PME) [32] was utilized to consider the long-range electrostatic interactions and the Linear Constraint Solver (LINCS) [33] algorithm was used to constrain bonds. The receptor–ligand complexes were solvated in a dodecahedron box of water, with a distance of 1.0 between the solute and the box. All systems were neutralized by adding Na^+^ and Cl^−^ at 0.15 mol/L. Before MD simulations, the complexes were relaxed to <1000 kJ/mol·nm by up to 50,000 cycles of steep descent minimization. After energy minimization, temperature of the system was controlled in the constant number of particles, volume, and temperature (NVT) ensemble to 300 K over 100 ps. The 100 ps constant number of particles, pressure, and temperature (NPT) equilibration was then performed with a reference pressure of 1 bar. After that, 30 ns MD simulations were performed with a time step of 2 fs and the coordinates of the complexes were saved every 8 ps.

### 3.4. Root-Mean-Square Fluctuation and Root-Mean-Square Deviation Analysis

The RMSF, RMSD, and secondary structure elements were analyzed by g_rmsf, g_rmsd, and do_dssp modules of the GROMACS 4.6.7 suite [29].

### 3.5. Binding Energy Calculation and Energy Decomposition

Molecular dynamics trajectory visualization was performed with VMD 1.9.2 [34]. The g_mmpbsa module of GROMACS 4.6.7 and mm_pbsa.pl tool of Amber 9 (University of California, San Francisco, CA, USA) were applied to calculate the free energies in biomolecular interactions.

The MM/PBSA was used to calculate the binding free energy of ALK–ligand complexes, as described in our previous study [35]. In this approach, the binding free energy (Δ*G*) calculation formula is [36]:

(1) $$\Delta G=\Delta E_{\mathrm{MM}}+\Delta G_{\mathrm{sol}}-T\Delta S$$

(2) $$\Delta E_{\mathrm{MM}}=\Delta E_{\mathrm{bonded}}+\left(\Delta E_{\mathrm{vdw}}+\Delta E_{\mathrm{ele}}\right)$$

(3) $$\Delta G_{\mathrm{sol}}=\Delta G_{\mathrm{polar}}+\Delta G_{\mathrm{nonpolar}}$$

The Van der Waals (Δ*E*_vdw_) and electrostatic interaction (Δ*E*_ele_) can be calculated through molecular mechanics (MM) method (Equation (2)). The bonded interactions (Δ*E*_bonded_) consisted of bond, angle, dihedral, and improper interactions. In the single trajectory approach, Δ*E*_bonded_ is always taken as zero because the conformations of protein and ligand are identified as a constant before and after they bond together [37]. The free energy of salvation (Δ*G*_sol_) consists of the polar solvation free energy and the nonpolar part which can be estimated with the Poisson–Boltzmann (PB) equation and the solvent-accessible surface area (SASA). *−T*Δ*S* stands for the changing of conformational entropy upon ligand binding, which usually is ignored in practice because of its high computational cost and low prediction accuracy.

In our research, the binding free energy by MM/GBSA is the sum of the gas-phase interaction energy (Δ*E*_MM_) plus the solvation free energy (Δ*G*_sol_) minus the product of the absolute temperature and entropy change (*T*Δ*S*) (Equation (4)).

(4) $$\Delta G=\Delta E_{\mathrm{MM}}+\Delta G_{\mathrm{sol}}-T\Delta S$$

(5) $$\Delta E_{\mathrm{MM}}=\Delta E_{\mathrm{vdw}}+\Delta E_{\mathrm{ele}}$$

(6) $$\Delta E_{\mathrm{sol}}=\Delta E_{\mathrm{GB}}+\Delta E_{\mathrm{SUR}}$$

The gas-phase interaction energy (Δ*E*_MM_) was composed of the van der Waals (Δ*E*_vdw_) and electrostatic interaction (Δ*E*_ele_) energies between receptor and ligand, and the solvation free energy (Δ*G*_sol_) was the sum of the electrostatic contributions calculated by the generalized Born (GB) approximation model (*E*_GB_) and the nonpolar contributions obtained using the SASA (*E_SUR_*). The entropic contribution (*T*Δ*S*) was ignored as it is computationally expensive to calculate and yields a low prediction accuracy. These terms were calculated using Equations (5) and (6).

### 3.6. Calculation of the Electrostatic Energy and Root-Mean-Square Fluctuation Change Trends

If the energy change from ALK–crizotinib to ALK–lorlatinib of the same amino acid residue was the same between 5–10 ns and 20–25 ns simulation, the residue received a score of 1. If the energy change trend was opposite, the residue received a score of −1. The trend between 20–25 ns and 25–30 ns was scored in the same way. The total energy change trend parameter was calculated by adding the score of the two-time periods together. A value of “2” suggested that the energy was changing in the same trend from ALK–crizotinib to ALK–lorlatinib in the investigated periods of time. The RMSF change trend of the key amino acids was analyzed in the same way as described above.

## 4. Conclusions

Acquired resistance to small molecule inhibitors is now a great challenge in the treatment of NSCLC and many other types of cancer. It is of great value to understand the molecular mechanism resulting in the occurrence of such resistance, which will in turn help the development of more specific and potent drugs to treat the disease. In this study, we exploited molecular dynamics simulation approach to understand how the lorlatinib ALK resistance mutation L1198F led to the resensitization to the first-generation inhibitor crizotinib. We found that crizotinib bound to C1156Y-L1198F ALK with a higher affinity than lorlatinib. This difference is at least partially caused by the conformational change that resulted from the L1198F mutation, as revealed by the RMSD analysis. By energy decomposition, we identified five amino acid residues that are located in the inhibitor and ATP binding domains of ALK. Root-mean-square fluctuation analysis further confirmed the importance of His1124 and Glu1210 as key amino acid residues that regulate the activity of ALK. We also found that Tyr1278 and Arg1279, located in the activation loop of the kinase domain, were also affected by the L1198 mutation. With all these results, we concluded that the L1198F mutation led to conformational changes at the inhibitor and ATP binding sites of ALK protein, which affected the downstream phosphorylation of the key residues. In clinical practice, it is very rare to see a resistance point mutation result in resensitization to the previous generation of inhibitors. In this study, we elucidated the molecular mechanism of such an intriguing drug sensitivity shift. These findings are valuable to the design of new targeted therapies for the treatment of ALK-positive cancer.

## Figures and Tables

**Table 1:** Distance analysis of inhibitor to hydrogen bonds forming amino acids of ALK.

	Distance to the Inhibitor (nm)							
	ALK–Crizotinib				ALK–Lorlatinib			
	0 ns	5–10 ns	20–25 ns	25–30 ns	0 ns	5–10 ns	20–25 ns	25–30 ns
Glu1197	3.02	2.53	2.68	2.66	2.81	2.94	3.02	3.02
Met1199	2.96	2.81	2.84	2.74	2.96	2.60	2.70	2.84

**Table 2:** Experimentally identified critical amino acid resides of ALK.

Residue	Described Function	References
His1124	ATP binding	[24,25]
Lys1150	Inhibitor binding	[24,26]
Met1199	Inhibitor binding	[24,26]
Asp1203	Inhibitor binding	[24,26]
Glu1210	Inhibitor binding	[24,26]
Tyr1278	Phosphorylation	[27]

## Supplementary Materials

Supplementary materials can be found at www.mdpi.com/1422-0067/18/3/482/s1.
