# Supplementary material for: L1198F Mutation Resensitizes Crizotinib to ALK by Altering the Conformation of Inhibitor and ATP Binding Sites
Source: Int J Mol Sci. 2017 Feb 24;18(3):482. doi: 10.3390/ijms18030482 (PMC5372498; doi:10.3390/ijms18030482)
Supplement: Supplementary file 1 [file ijms-18-00482-s001.zip › ijms-174979_supplementary_final.pdf]

# Supplementary Materials: L1198F Mutation Resensitizes Crizotinib to ALK by Altering the Conformation of Inhibitor and ATP Binding Sites

**Jian Li, Rong Sun, Yuehong Wu, Mingzhu Song, Jia Li, Qianye Yang, Xiaoyi Chen, Jinku Bao and Qi Zhao**

**Table S1.** Predicted binding energy of anaplastic lymphoma kinase (ALK)–crizotinib and ALK–lorlatinib complexes.

| PDB ID | Mutation | Inhibitor | Ki | $IC_{50}$ | AUTODOCK (kJ/mol) | Hawkins_GB/SA (kJ/mol) | MM/PBSA (kJ/mol) |
|---|---|---|---|---|---|---|---|
| 5AA8 | C1156Y L1198F | Lorlatinib | 61 | 67 | 123.7 | −77.44 | −26.66 |
| 5AAB | C1156Y L1198F | Crizotinib | 0.6 | 0.6 | 25.9 | −472.74 | −231.88 |
| Folds of change (crizotinib/lorlatinib) | C1156Y L1198F | - | 111.7 | 111.7 | 4.8 | 6.1 | 8.7 |

GB/SA: Generalized Born surface area; MM/PBSA: Molecular mechanics Poisson–Boltzmann surface area; PDB: Protein Data Bank.

**Table S3.** Key amino acid residues contributing to the electrostatic energy.

| Position | PDB ID | Electrostatic Energy (kcal/mol) | | | Folds of Change | | | Average |
|---|---|---|---|---|---|---|---|---|
| | | 5–10 ns | 20–25 ns | 25–30 ns | 5–10 ns | 20–25 ns | 25–30 ns | |
| 1124 | 5AA8 | −10.74 | −10.98 | −10.90 | −10.90 | 6.73 | 5.74 | 5.60 |
| | 5AAb | −46.58 | −73.95 | −62.56 | | | | |
| 1150 | 5AA8 | −79.38 | −69.78 | −72.11 | 1.24 | 1.08 | 1.09 | 1.14 |
| | 5AAb | −64.01 | −64.55 | −66.09 | | | | |
| 1119 | 5AA8 | −7.22 | −8.98 | −8.61 | 1.26 | 1.07 | 1.07 | 1.14 |
| | 5AAb | −9.1 | −9.63 | −9.25 | | | | |
| 1203 | 5AA8 | −1.67 | −5.4 | −2.28 | 11.63 | 3.68 | 8.28 | 7.86 |
| | 5AAb | −19.42 | −19.88 | −18.87 | | | | |
| 1210 | 5AA8 | 4.65 | 13.43 | 16.47 | 2.74 | 3.82 | 24.58 | 8.55 |
| | 5AAb | −12.73 | 3.52 | 0.67 | | | | |

**Table S4.** Trends of root-mean-square fluctuation (RMSF) change of the key amino acid residues in ALK.

| Position | PDB ID | RMSF (nm) | | | Interval Coefficient | | RMSF Coefficient | Relative Folds of Change | | | Average |
|---|---|---|---|---|---|---|---|---|---|---|---|
| | | 5–10 ns | 20–25 ns | 25–30 ns | 5-10 ns/20–25 ns | 20–25 ns/25–30 ns | | 5–10 ns | 20–25 ns | 25–30 ns | |
| 1270 | 5AA8 | 0.0413 | 0.0365 | 0.036 | −1 | 1 | 0 | | | | |
| | 5AAB | 0.0401 | 0.0376 | 0.0397 | | | | | | | |
| 1124 | 5AA8 | 0.1004 | 0.102 | 0.0985 | 1 | 1 | 2 | 1.53 | 2.34 | 1.53 | 1.80 |
| | 5AAB | 0.1538 | 0.2382 | 0.1511 | | | | | | | |
| 1150 | 5AA8 | 0.0496 | 0.0496 | 0.0461 | 1 | −1 | −2 | | | | |
| | 5AAB | 0.0483 | 0.0462 | 0.051 | | | | | | | |
| 1119 | 5AA8 | 0.0512 | 0.0455 | 0.0548 | −1 | −1 | 0 | | | | |
| | 5AAB | 0.0501 | 0.0591 | 0.0414 | | | | | | | |
| 1203 | 5AA8 | 0.0405 | 0.042 | 0.0448 | 1 | −1 | 2 | | | | |
| | 5AAB | 0.0462 | 0.0537 | 0.0422 | | | | | | | |
| 1210 | 5AA8 | 0.0652 | 0.0612 | 0.0544 | 1 | 1 | 0 | 1.04 | 1.67 | 1.16 | 1.29 |
| | 5AAB | 0.0678 | 0.1023 | 0.063 | | | | | | | |
| 1249 | 5AA8 | 0.0412 | 0.0393 | 0.0367 | −1 | 1 | 0 | | | | |
| | 5AAB | 0.0366 | 0.0433 | 0.0384 | | | | | | | |
| 1278 | 5AA8 | 0.0812 | 0.0739 | 0.0781 | −1 | 1 | 0 | | | | |
| | 5AAB | 0.0773 | 0.1089 | 0.088 | | | | | | | |
| 1279 | 5AA8 | 0.1008 | 0.098 | 0.0845 | −1 | 1 | 0 | | | | |
| | 5AAB | 0.0915 | 0.1336 | 0.113 | | | | | | | |
